# Integrated analysis of hematopoietic differentiation outcomes and molecular characterization reveals unbiased differentiation capacity and minor transcriptional memory in HPC/HSC-iPSCs

**DOI:** 10.1186/s13287-016-0466-1

**Published:** 2017-01-23

**Authors:** Shuai Gao, Xinfeng Hou, Yonghua Jiang, Zijian Xu, Tao Cai, Jiajie Chen, Gang Chang

**Affiliations:** 1 School of Medicine, Shenzhen University, Nanhai Ave 3688, Shenzhen, 518060 Guangdong China; 2 National Institute of Biological Sciences, NIBS, Beijing, 102206 China

**Keywords:** Hematopoietic progenitor and stem cells, Induced pluripotent stem cells, Transcriptional memory, Differentiation

## Abstract

**Background:**

Transcription factor-mediated reprogramming can reset the epigenetics of somatic cells into a pluripotency compatible state. Recent studies show that induced pluripotent stem cells (iPSCs) always inherit starting cell-specific characteristics, called epigenetic memory, which may be advantageous, as directed differentiation into specific cell types is still challenging; however, it also may be unpredictable when uncontrollable differentiation occurs. In consideration of biosafety in disease modeling and personalized medicine, the availability of high-quality iPSCs which lack a biased differentiation capacity and somatic memory could be indispensable.

**Methods:**

Herein, we evaluate the hematopoietic differentiation capacity and somatic memory state of hematopoietic progenitor and stem cell (HPC/HSC)-derived-iPSCs (HPC/HSC-iPSCs) using a previously established sequential reprogramming system.

**Results:**

We found that HPC/HSCs are amenable to being reprogrammed into iPSCs with unbiased differentiation capacity to hematopoietic progenitors and mature hematopoietic cells. Genome-wide analyses revealed that no global epigenetic memory was detectable in HPC/HSC-iPSCs, but only a minor transcriptional memory of HPC/HSCs existed in a specific tetraploid complementation (4 N)-incompetent HPC/HSC-iPSC line. However, the observed minor transcriptional memory had no influence on the hematopoietic differentiation capacity, indicating the reprogramming of the HPC/HSCs was nearly complete. Further analysis revealed the correlation of minor transcriptional memory with the aberrant distribution of H3K27me3.

**Conclusions:**

This work provides a comprehensive framework for obtaining high-quality iPSCs from HPC/HSCs with unbiased hematopoietic differentiation capacity and minor transcriptional memory.

**Electronic supplementary material:**

The online version of this article (doi:10.1186/s13287-016-0466-1) contains supplementary material, which is available to authorized users.

## Background

Adult cells can be reverted into a pluripotent state by forcing ectopic expression of a set of transcription factors, and the resulting induced pluripotent stem cells (iPSCs) demonstrate differentiation potential into all three germ layers [1]. iPSCs can be maintained at the pluripotent state under defined culture conditions, holding great promise for regenerative medicine. However, the reprogramming efficiency is always very low [2], indicating that reprogramming is a complicated process. A recent study has revealed a detailed route map of reprogramming, demonstrating that reprogramming is not simply the reversal of the normal developmental process [3]. Previous studies have revealed that the generation of iPSCs is accompanied by a phenomenon called epigenetic memory, and that the resulting iPSCs often inherit the epigenetic characteristics of the starting cells and display a tendency to differentiate toward the original cell lineage [4–7]. Similar results were obtained in another study, in which incomplete reprogramming was discovered to be associated with aberrant epigenetic modifications [8]. Compared with other pluripotent stem cells, iPSCs retained more residual DNA methylation patterns of parental somatic cells [9]. In terms of biosafety concerns, the safety issues surrounding epigenetic memory in reprogramming are not easy to predict. It is noteworthy that the process of induced reprogramming always coincided with the appearance of cancer-related epigenetic abnormalities [10]. Therefore, obtaining iPSCs with an unbiased differentiation capacity and no somatic memory, and further characterization of the underlying mechanisms of such reprogramming is important for the therapeutic use of such cells.

Hematopoietic cells have been widely used in transcription factor-mediated reprogramming studies as they are convenient to harvest [11, 12]. The well-defined hematopoiesis hierarchy provides more information for the derivation of hematopoietic cells from progenitor cells or pluripotent cells, and it also works as a good standard for evaluating the differentiation potential of iPSCs [13]. Notably, epigenetic memory was first discovered in the reprogramming of hematopoietic and mesenchymal lineage cells [4]. Among hematopoietic populations, hematopoietic progenitor and stem cells (HPC/HSCs) have proven to be susceptible to transcription factor-mediated reprogramming with high efficiency [13, 14]. Similarly, our previous study revealed that HPC/HSCs are amenable to reprogramming, with a resulting high frequency of high-quality HPC/HSC-iPSCs with full pluripotency [15]. Therefore, it is of interest to test whether HPC/HSC-iPSCs manifest other outstanding characteristics related to their differentiation capacity or novel molecular features.

In the present study, we aim to evaluate the differentiation capacity and molecular features of HPC/HSC-iPSCs under a previously established sequential reprogramming system [16]. Our results reveal that HPC/HSCs are a novel cell resource for obtaining iPSCs without biased differentiation and epigenetic memory.

## Methods

### Cell culture

All embryonic stem cells (ESCs) and iPSCs were cultured on mitomycin C-treated (Sigma-Aldrich, St. Louis, MO, USA) mouse embryonic fibroblasts (MEFs) in ES medium, which consisted of Dulbecco modified Eagle’s medium (Invitrogen, Carlsbad, CA, USA) supplemented with 15% fetal bovine serum (FBS; Hyclone, South Logan, UT, USA), 1 mM L-glutamine (Invitrogen), 0.1 mM β-mercaptoethanol (Invitrogen), 1% nonessential amino acid (Invitrogen) and 1000 U/mL leukemia inhibitory factor (EMD Millipore, Billerica, MA, USA).

### Western blotting

RIPA buffer was used to prepare the cell extracts. Samples were resolved on SDS-PAGE gels, and transferred to polyvinylidene fluoride membranes (BioRad. Hercules, CA, USA). Specific proteins were further analyzed using anti-Oct3/4 (Santa Cruz Biotechnology, Dallas, TX, USA), anti-Sox2 (Abcam, Cambridge, UK), anti-Klf4 (R&D Systems, Minneapolis, MN, USA), anti-c-Myc (Abcam) and anti-Gapdh (Sigma-Aldrich) antibodies. Peroxidase-labeled anti-mouse, rabbit or goat antibodies labeled with enhanced chemiluminescence (GE Healthcare, Chicago, IL, USA) were used for further detection.

### Hematopoietic differentiation of iPSCs and ESCs

An OP9 co-culture assay was performed as previously described [17]. OP9 stromal cells were plated onto gelatinized 100-mm dishes in α-MEM (Invitrogen) containing 20% FBS (Hyclone), 2 mM L-glutamax (Invitrogen) and 1% penicillin/streptomycin (EMD Millipore). Feeder-free ESCs or iPSCs were replated in dishes where OP9 cells had formed confluent cultures on day 6 at a density of 1.5 × 10^6^ per 100-mm dish in α-MEM (Invitrogen) supplemented with 10% FBS (HyClone) and 100 μM monothioglycerol (MTG; Sigma-Aldrich). The co-cultured cells were incubated for another 8 days with a half-medium change every other day. Single-cell suspensions were prepared for flow-cytometry analysis on day 8.

A hematopoietic colony-forming assay was performed using a standard kit (Stemcell Technologies, Vancouver, BC, USA). In brief, ESCs and iPSCs were cultured on gelatin-coated dishes for two passages to remove MEFs prior to embryonic body (EB) differentiation, and feeder-free ESCs and iPSCs were harvested by centrifugation at 200 × *g* for 5 min. The cell pellet was resuspended in differentiation medium, containing 15% FBS, 1% penicillin/streptomycin, 1% L-glutamine (Invitrogen), 150 μM MTG (Sigma-Aldrich), 100 μM ascorbic acid-vitamin C (Sigma-Aldrich) and hematopoietic growth factors at a density of 4 × 10^5^/25 mL. To form EBs, the cells in “Reagent Reservoirs” were then transferred to the covers of 15-cm dishes using a multichannel pipette; each drop had a volume of 25 μL. Four days later, EBs were collected from the hanging drops and transferred to an ultra-low attachment dish to continue the differentiation process. Hematopoietic colony assays were performed using 50,000 D6 EB cells in hematopoietic differentiation medium. Colony-forming units (CFUs) were counted from three experiments for erythrocytes (BFU-E), macrophages (M), granulocyte-macrophages (GM) and granulocyte-erythrocyte-macrophage-megakaryocytes (GEMM) after 10–14 days of culture. The medium and additional components used here are all listed in the Stemcell Technologies technical manual.

### Flow cytometry analysis

To analyze the differentiation capacity to hematopoietic progenitors, ESCs/OP9 and iPSCs/OP9 co-cultured cells were collected and washed using fluorescence-activating cell sorting (FACS) buffer (phosphate-buffered salt solution with 2% FBS). Cells were then stained using an anti-mouse SSEA-1-Alexa Fluor 647 monoclonal antibody (eBioscience, San Diego, CA, USA), an anti-mouse c-Kit-APC monoclonal antibody (eBioscience), an anti-mouse Sca-1-PE/Cy7 monoclonal antibody (eBioscience) and an anti-mouse CD34-Alexa Fluor PE monoclonal antibody (eBioscience) in different combinations. After the staining, cells were washed and resuspended in FACS buffer. Gating was performed using matched isotype control monoclonal antibodies. Fluorescent-activated cell analyses were performed using a MoFlo XDP cell sorter (Beckman Coulter Inc., Brea, CA, USA).

For the analyses of ESCs/OP9 and iPSCs/OP9 co-cultures, the differentiated states of ESCs or iPSCs were first analyzed according to the expression of SSEA-1 on day 8. Then, cells with large diameters were removed first and GFP-positive cells (OP9) were removed next. Finally, c-Kit^+^Sca-1^+^ double-positive cells were divided into two subpopulations for the analysis of long-term and short-term HSCs based on the expression of CD34.

### RNA sequencing (RNA-Seq), methylated DNA immunoprecipitation sequencing (MeDIP-Seq) and chromatin immunoprecipitation sequencing (ChIP-Seq)

The sample preparation, library generation, and sequence analysis of RNA-Seq, MeDIP-Seq, and ChIP-Seq were all in accordance with our previously reported methods [18]. In general, the sequencing was performed at the Beijing Genomics Institute using the HiSeq 2000 system (Illumina, San Diego, CA, USA). Single-end sequencing was applied to RNA-Seq and ChIP-Seq. Paired-end sequencing was applied to MeDIP-Seq.

### Bioinformatics analyses

The MeDIP/ChIP-Seq reads were mapped to the mouse reference genome (mm9/NCBI37) using Bowtie (v0.12.7) allowing the max mismatch of 3 nt [19]. The RNA-Seq reads were mapped to the mouse genome using Tophat (v1.3.3) and the Ensembl genome annotation (Mus_musculus.NCBIM37.64.gtf) [20]. Cufflink (v1.2.0) software was used to analyze the mRNA expression patterns (FPKM, fragments per kilobase of exon per million fragments mapped) as previously described [21]. MACS (v1.4.1) software was used to identify regions enriched for DNA methylation (peaks) using the default parameters [22]; the cutoff *P* value was set at 1 × 10^-5^. CCAT (v3.0) software was used to identify histone modification-enriched regions (peaks) using the parameters as previously reported [23]; the FDR cutoff was set at 0.05.

For the transcriptional memory analysis in HPC/HSC-iPSCs, we first identified genes specifically expressed by HPC/HSCs using two cell types as controls. The genes with low expression were filtered out using a standard of FPKM ≥10 in at least one sample. ANOVA was then applied to identify genes that were significantly differentially expressed in the somatic samples. HPC/HSC-specific genes were extracted only if the FPKM values of the genes were twofold higher or lower in HPC/HSCs than in any other cell line. Genes that were differentially expressed in HPC/HSC-iPSC lines were then identified and compared with HPC/HSC-specific genes. The overlapping gene sets were analyzed using Gene Ontology (GO) tools (DAVID) to identify enriched GO terms [24]. Normalized 5mC, H3K4me2, H3K4me3, and H3K27me3 read counts in the transcription start site (TSS) ± 2 kb regions of different gene sets were represented using the R boxplot function.

### Statistical analyses

Data was analyzed by the GraphPad Prism5 (GraphPad Software, Inc, La Jolla, CA, USA). The experiments were repeated independently three times. Statistical differences were evaluated using the Student’s *t* test as indicated. Standard deviation (SD) was used to assess biological significance.

### Accession numbers

The GEO accession number for the genome-wide sequencing data reported in this article is GSE36294.

## Results

### Unique histone modification patterns in HPC/HSC-specific genes and the stoichiometry of transcription factors in the reprogramming of HPC/HSCs

**Fig. 1:**
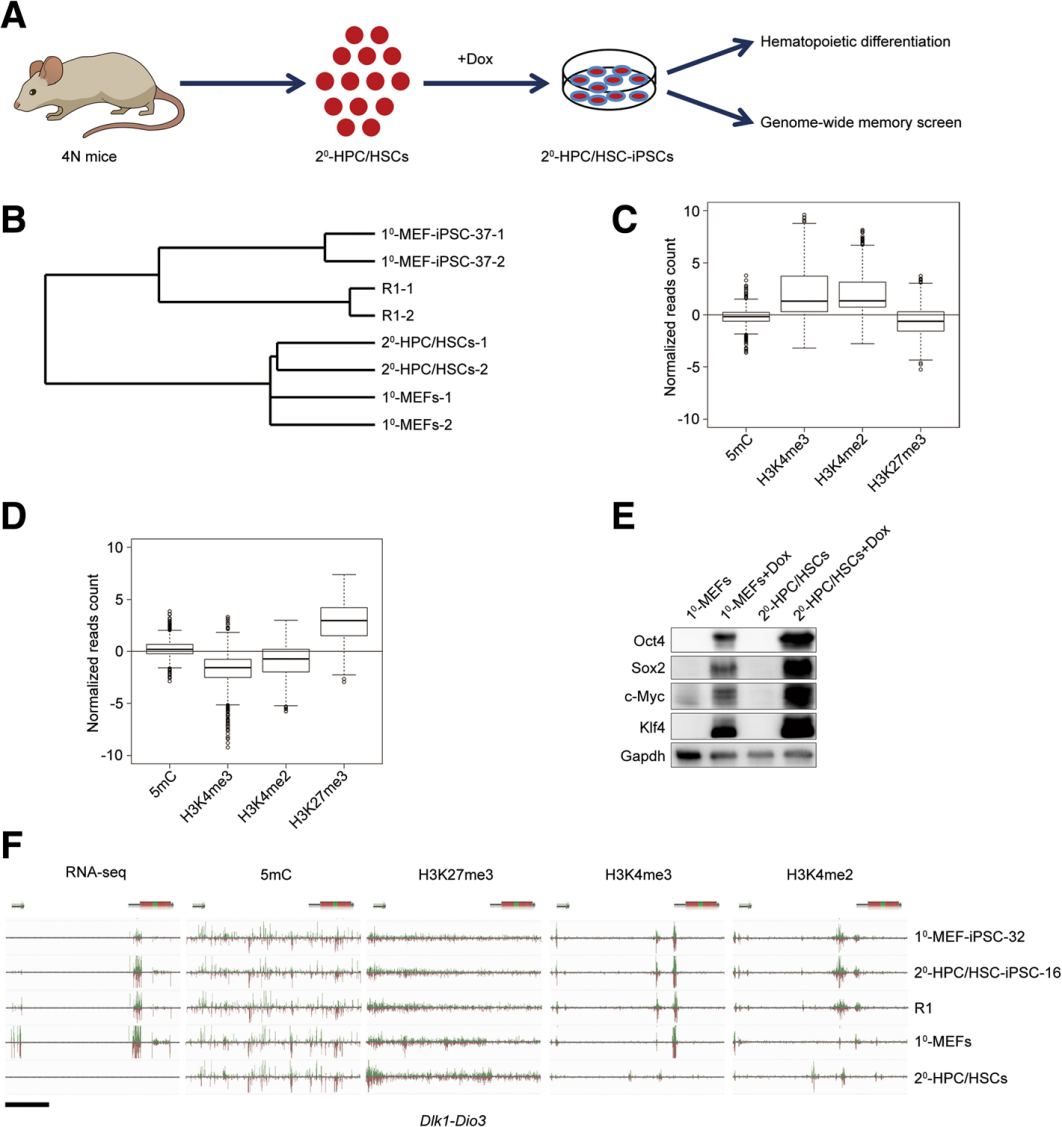
Genome-wide cellular characteristics of HPC/HSCs and OSKM-mediated reprogramming of HPC/HSCs. **a** Schematic showing the generation of HPC/HSC-iPSCs and the evaluation of hematopoietic differentiation potential and genome-wide memory status. **b** Hierarchical clustering of genome-wide gene expression patterns of HPC/HSCs, MEFs, R1 ESCs and iPSCs. The analysis of each cell line was repeated twice. **c** Distribution of DNA methylation (5mC) and histone modifications (H3K4me2, H3K4me3, and H3K27me3) in the TSS ± 2 kb regions of genes with high expression in HPC/HSCs. The normalized read counts in HPC/HSCs minus the counts in MEFs were used for the plots. **d** Distribution of DNA methylation (5mC) and histone modifications (H3K4me2, H3K4me3, and H3K27me3) in the TSS ± 2 kb regions of genes with low expression in HPC/HSCs. The normalized read counts in HPC/HSCs minus the counts in MEFs were used for the plots. **e** Western blot comparing the expression levels of the four reprogramming factors in OSKM-mediated reprogramming in the isolates of HPC/HSCs and MEFs. *4 N* tetraploid complementation, *HPC/HSC* hematopoietic progenitor and stem cell, *iPSC* induced pluripotent stem cell, *MEF* mouse embryonic fibroblast, *RNA-Seq* RNA sequencing. **f** The read distribution of RNA-Seq, H3K27me3, H3K4me3, H3K4me2 ChIP-Seq and DNA methylation (5mC) in the region corresponding to the upstream region of the *Dlk1*-*Dio3* locus in R1, MEFs, and HPC/HSCs. Scale bar, 30 kb

Under the previously established sequential reprogramming system [16], HPC/HSCs were found to be amenable to reprogramming into high-quality iPSCs with full pluripotency [15]. It is also of interest to know whether the reprogramming of HPC/HSCs could give rise to iPSCs with unbiased differentiation and no somatic memory. To this aim, integrated analysis of hematopoietic differentiation outcomes and molecular characterization of HPC/HSC-iPSCs was performed in the present study (Fig. 1a).

The epigenetic memory of iPSCs detailed in previous studies has always been associated with the expression of starting cell-specific genes [4, 5]. Therefore, it is of interest to understand the molecular features of HPC/HSC-specific genes, which may determine the reprogramming process and the differentiation capacity of HPC/HSC-iPSCs. First, global gene expression patterns were compared among HPC/HSCs, MEFs, R1, and iPSCs (1^0^-MEF-iPSC-37). Hierarchical clustering analyses show that HPC/HSCs have differential gene expression patterns compared with MEFs and pluripotent stem cells (Fig. 1b). HPC/HSC-specific genes were screened out by comparing the gene expression patterns between HPC/HSCs and MEFs. To investigate the regulatory mechanisms underlying the differential expression of these genes, high-throughput sequencing was performed to investigate the DNA methylation (5mC) and core histone modifications (H3K4me2, H3K4me3, and H3K27me3) near the TSS of the HPC/HSC-specific genes. We found that DNA methylation was not associated with the expression of these two gene groups (Fig. 1c, d). However, H3K4me3 and H3K4me2 were found to be enriched in the TSS of the highly expressed HPC/HSC genes but absent from that of the lowly expressed HPC/HSC genes. However, H3K27me3 showed a contrasting pattern, being enriched in the TSS of highly expressed HPC/HSC genes but absent from that of lowly expressed HPC/HSC genes (Fig. 1c, d). Interestingly, the presence or absence of H3K4me3 and H3K4me2 was synchronous for the TSS of most differentially expressed genes. Therefore, the observed reciprocal occupancy of H3K4me2/H3K4me3 and H3K27me3 may inversely regulate the expression of HPC/HSC-specific genes, which is in contrast to the bivalent histone modification profiles found in embryonic stem cells [25]. In summary, our results show that balanced occupancy of H3K4me3/H3K4me2 and H3K27me3 may play an important role in controlling the expression patterns of HPC/HSC-specific genes.

Another feature of HPC/HSC reprogramming is the high frequency of iPSCs with full pluripotency, as we previously reported [15]. It has been demonstrated that the stoichiometry of reprogramming factors has a great influence on the quality of iPSCs [26]. Therefore, the stoichiometry of transcription factors during the reprogramming process of HPC/HSCs was compared, and it was found that intermediate cells of the HPC/HSC reprogramming showed higher expression of Oct4, Sox2, Klf4, and c-Myc than their counterparts during MEF reprogramming (Fig. 1e). Of note, differential transcription factor stoichiometry was observed only during the reprogramming process but not in the starting cells, demonstrating that the roadmap of HPC/HSC reprogramming is unique compared with that of MEFs.

To test whether the observed particular core histone modifications (H3K4me3, H3K4me2, and H3K27me3) occupancy patterns exist in specific gene loci, we examined the epigenetic modifications of *Dlk1-Dio3*, which was found to be associated with the pluripotency state of iPSCs [27, 28]. The results show that the RNA-Seq profile at the *Dlk1-Dio3* locus of HPC/HSCs is dramatically different from that of MEFs (Fig. 1f). In accordance with genome-wide similarities of DNA methylation, the distribution of the 5mC signal at this locus showed no difference between HPC/HSCs and MEFs (Fig. 1f). However, core histone modifications (H3K4me2, H3K4me3 and H3K27me3) were found to be differentially distributed at the *Dlk1-Dio3* locus between HPC/HSCs and MEFs. Notably, the absence of RNA-Seq read distribution in the HPC/HSCs corresponded to the enrichment of H3K27me3 and the decrease of H3K4me3, which was generally consistent with the previously discussed global core histone modifications (H3K4me2, H3K4me3, and H3K27me3) patterns in HPC/HSC-specific genes. In addition, the core histone modifications (H3K4me2, H3K4me3, and H3K27me3) in both HPC/HSCs and MEFs were distinguishable from that of iPSCs and R1, indicating that the starting cells from different origins undergo distinct epigenetic reprogramming to achieve a pluripotent state. In support of this result, a recent study that re-interpreted our small RNA sequencing dataset [18] revealed a unique reprogramming process of HPC/HSCs, in which some lncRNAs were distinctively activated to repress HPC/HSC-lineage-specific genes [29].

Together, HPC/HSC-specific genes were found be modified with unique reciprocal occupancy of H3K4me2/H3K4me3 and H3K27me3. The reprogramming process of HPC/HSCs demonstrates a specific stoichiometry of transcription factors, in which Oct4, Sox2, Klf4, and c-Myc are highly expressed compared with that of MEFs.

### Unbiased hematopoietic differentiation capacity of HPC/HSC-iPSCs

As mentioned above, core histone modifications (H3K4me2, H3K4me3, and H3K27me3) patterns in HPC/HSC-iPSCs and the transcription factor stoichiometry in HPC/HSC reprogramming were examined. However, it is unknown whether these molecular features could facilitate the overcoming of reprogramming barriers to achieve complete reprogramming, which may result in iPSCs with unbiased differentiation capacity and no somatic memory of the starting cells. Recent findings have suggested that hematopoietic cell-derived iPSCs retain a biased differentiation tendency toward the cell lineage of their starting cells, which results from epigenetic memory [4, 5]. To assess the differentiation capacity of HPC/HSC-iPSCs, in vitro hematopoietic differentiation was performed in the four HPC/HSC-iPSC lines, five mesenchyme-derived iPSC lines (M-iPSCs) and one ESC line (R1). To eliminate the influence of continuous passaging of iPSCs on their epigenetic memory, the iPSC lines used for the differentiation assays here were all at an early passage (<p8) stage, which is consistent with previous studies [4, 5].

**Fig. 2:**
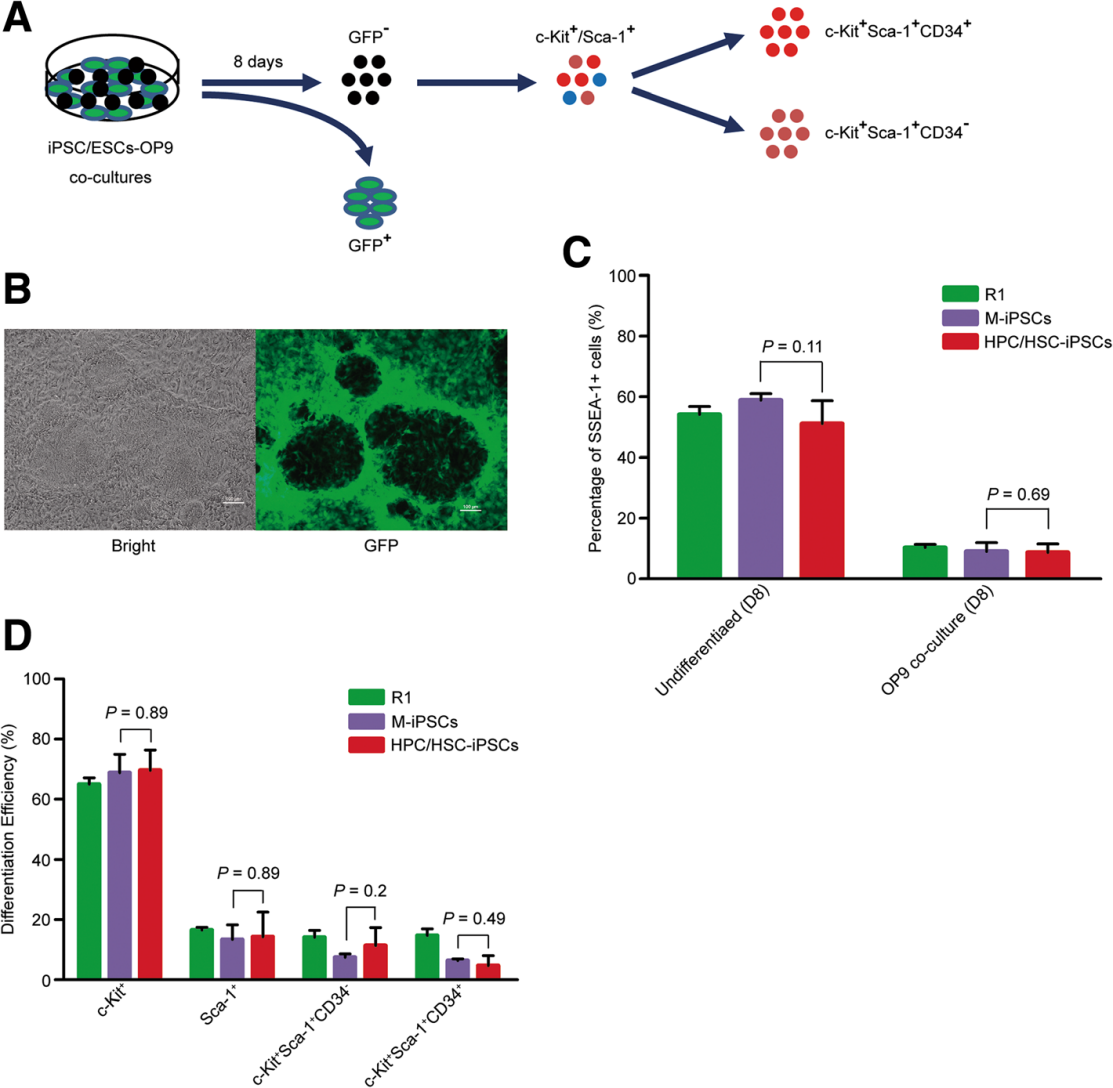
HPC/HSC-iPSCs showed unbiased differentiation potential into hematopoietic progenitor cells. **a** Schematic illustration of the flow cytometry analysis. **b** Typical cell morphology of ESCs and iPSCs co-cultured with OP9 stromal cells. *Left*, bright field; *GFP*, the field under fluorescence microscopy with an excitation peak of 488 nm. **c** Expression of SSEA-1 by ESCs and iPSCs as detected using flow cytometry after co-culture with OP9 stromal cells for 8 days. Error bars indicate the SD *P*, *P* value, unpaired *t* test. **d** Differentiation efficiency of ESCs and iPSCs into different types of hematopoietic progenitor cells; Error bars indicate the SD *P*, *P* value, unpaired *t* test. *HPC/HSC* hematopoietic progenitor and stem cell, *iPSC* induced pluripotent stem cell, *SD* standard deviation

An OP9 co-culture system was used to evaluate the differentiation capacity of HPC/HSC-iPSCs and M-iPSCs to hematopoietic cells, as previously described [17], and this was implemented using a hierarchical cell-sorting analysis during flow cytometry (Fig. 2a). In the OP9 co-culture system, typical differentiated iPSCs/ESCs could be easily distinguished from GFP-positive OP9 stromal cells (Fig. 2b). We found that HPC/HSC-iPSCs yielded a similar frequency of SSEA-1^+^ cells when cultured on mitomycin C-treated MEFs or 8 days after differentiation by co-culture with OP9 cells, compared with the frequency of SSEA-1^+^ M-iPSCs (Fig. 2c and Additional file 1: Figure S1). Hierarchical cell sorting was performed based on specific cell lineage markers, and the GFP-positive cells (OP9) were discarded first (Additional file 2: Figure S2). GFP-negative cells were maintained for further isolation of Sca-1^+^ and c-Kit^+^ cells. We found that similar proportions of Sca-1^+^ and c-Kit^+^ cells were differentiated from both HPC/HSC-iPSCs and M-iPSCs (Fig. 2d). Finally, c-Kit^+^Sca-1^+^ cells, which contained the HPC/HSCs were further analyzed and we detected comparable proportions of c-Kit^+^Sca-1^+^CD34^+^ cells (short-term HSCs) and c-Kit^+^Sca-1^+^CD34^-^ cells (long-term HSCs) from both the HPC/HSC-iPSCs and M-iPSCs (Fig. 2d). Therefore, HPC/HSC-iPSCs and M-iPSCs had a similar capacity to differentiate into progenitors of hematopoietic cells.

**Fig. 3:**
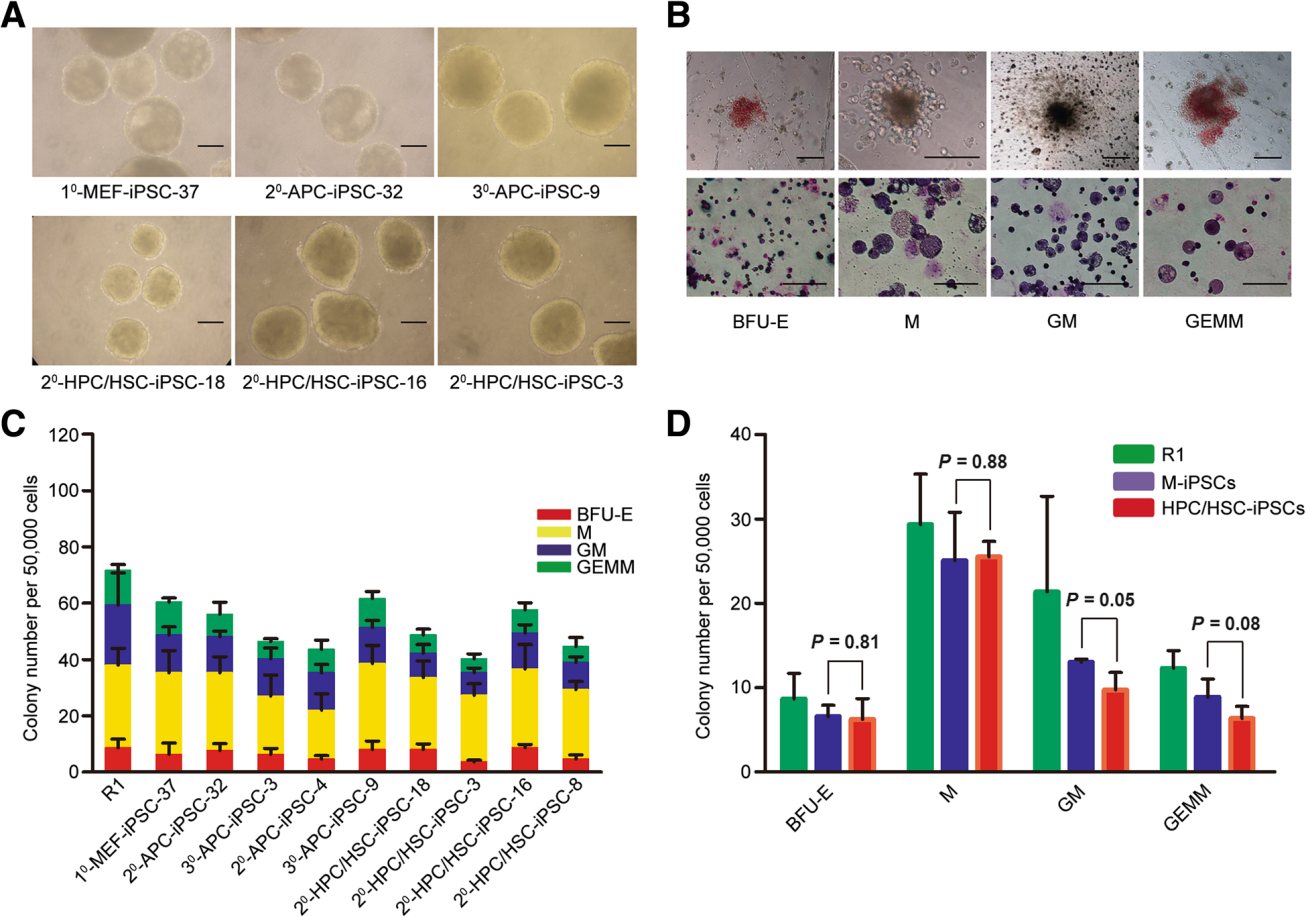
HPC/HSC-iPSCs showed unbiased differentiation potential into hematopoietic mature cells. **a** Embryonic bodies derived from early-passage mesenchyme-iPSCs and HPC/HSC-iPSCs; scale bars, 200 μm. **b** Representative hematopoietic colonies derived from iPSCs with Wright–Giemsa staining to reveal the hematopoietic progenitors. *From left to right*: *BFU-E* erythrocyte, *M* macrophage, *GM* granulocyte-macrophage, and *GEMM* granulocyte-erythrocyte-macrophage-megakaryocyte. Scale bars: *top*, 100 μm; *bottom*, 40 μm. **c** In vitro hematopoietic colony number per 50,000 cells of EBs differentiated from mesenchyme-iPSCs (n = 5) and hematopoietic-iPSCs (n = 4), using R1 cells as a control. The error bars indicate SD. **d** The comparison of CFU numbers between mesenchyme-iPSCs and hematopoietic-iPSCs (n = 3 measurements). Error bars indicate the SD *P*, *P* value, unpaired *t* test. *EB* embryonic body, *HPC/HSC* hematopoietic progenitor and stem cell, *iPSC* induced pluripotent stem cell, *CFU* colony-forming unit

To further test whether HPC/HSC-iPSCs preferentially differentiate into mature hematopoietic cells, hematopoietic colony-forming capacity was evaluated in HPC/HSC-iPSCs and M-iPSCs. First, EBs were generated from HPC/HSC-iPSCs and M-iPSCs, which had similar morphology and size (Fig. 3a). Then, the cells which dissociated from mature EBs were further differentiated in hematopoietic differentiation medium, and typical hematopoietic colonies could be observed (Fig. 3b). The results show that HPC/HSC-iPSCs and M-iPSCs yielded similar numbers of the four types of hematopoietic colonies (Fig. 3c, d). In summary, the in vitro hematopoietic differentiation results reveal that iPSCs derived from HPC/HSCs manifested an apparently unbiased differentiation capacity to both hematopoietic progenitor cells and mature hematopoietic cells compared with that of MEFs.

### Minor transcriptional memory retained in specific HPC/HSC-iPSCs

Our previous study demonstrated that a large proportion of HPC/HSC-iPSC lines manifest full pluripotency, which is only one symbol of complete reprogramming [15]. Based on the hematopoietic differentiation results described above, HPC/HSC-iPSCs have an unbiased differentiation capacity to both hematopoietic progenitor cells and mature hematopoietic cells. Therefore, the reprogramming of HPC/HSCs appears complete, and could enable the production of high-quality iPSCs with full pluripotency and an unbiased differentiation capacity. However, it remains unclear whether HPC/HSC-iPSCs retain a somatic memory state of their starting cells.

**Fig. 4:**
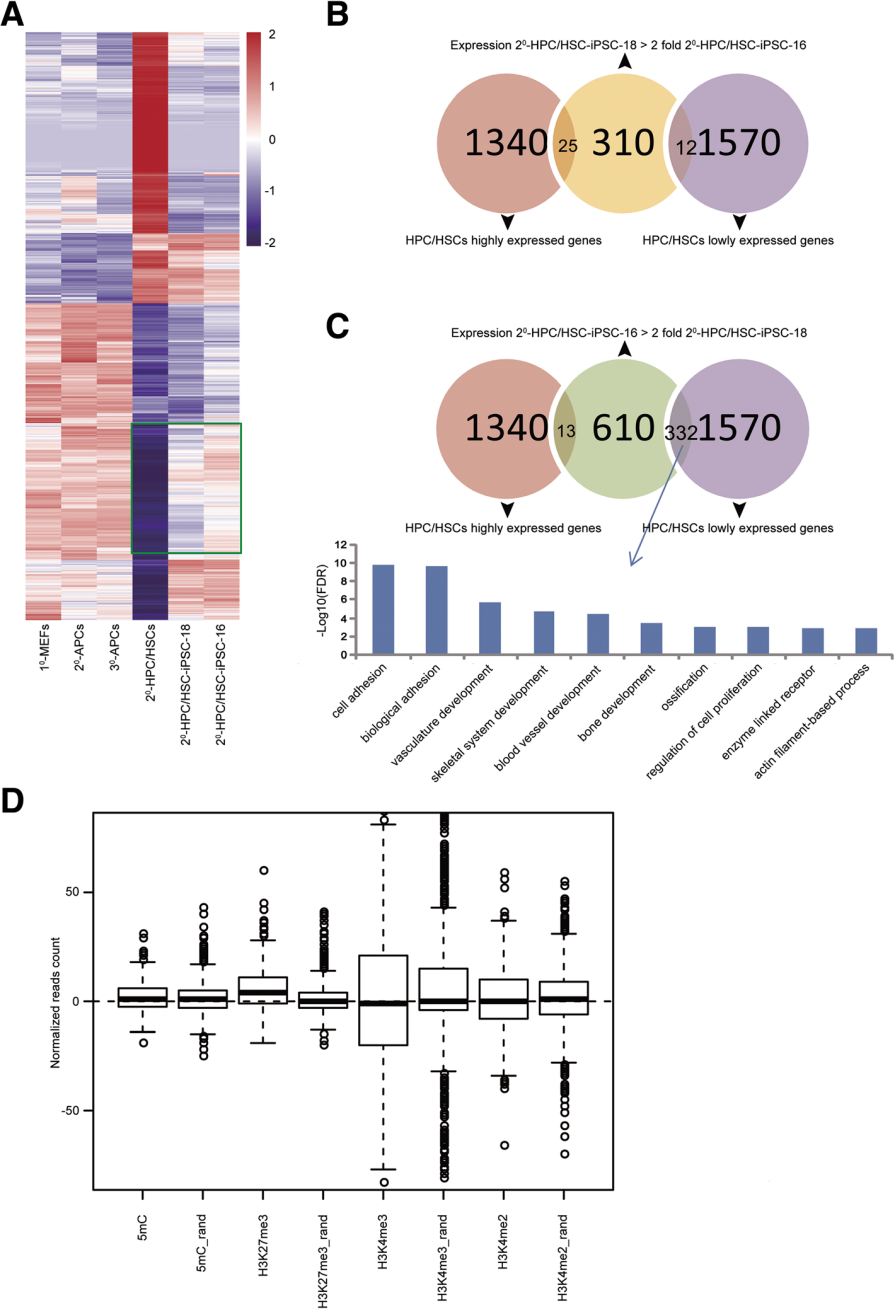
Minor transcriptional memory in specific HPC/HSC-iPSC lines. **a** The “4 N-OFF” 2^0^-HPC/HSC-iPSC-18 line is more similar to HPC/HSCs than the “4 N-ON” 2^0^-HPC/HSC-iPSC-16 line; note the similarity of its gene expression profile to that of the HPC/HSC genes with low expression. The *green box* marks genes that are differentially expressed in the 2^0^-HPC/HSC-iPSC-18 and 2^0^-HPC/HSC-iPSC-16 lines. **b** The number of highly expressed genes in the 2^0^-HPC/HSC-iPSC-18 line that overlapped with highly expressed genes in the HPC/HSCs was greater than the number that overlapped with genes with low expression in the HPC/HSCs*. Sky-blue*, HPC/HSC genes with low expression; *pink*, HPC/HSC genes with high expression; *orange*, greater than a twofold change between the two cell lines (18 > 16). **c** The number of highly expressed genes in the 2^0^-HPC/HSC-iPSC-16 line that overlapped with genes with low expression in the HPC/HSCs was greater than the overlap with the HPC/HSC highly expressed genes. The top ten most common enriched GO term categories represented by these genes are plotted. *Sky-blue*, HPC/HSC genes with low expression; *pink*, HPC/HSC genes with high expression; *pea green*, greater than a twofold change between the two cell lines (16 > 18). **d** Epigenetic modifications in the 332 overlapping genes in the 2^0^-HPC/HSC-iPSC-18 and 2^0^-HPC/HSC-iPSC-16 lines were compared. The distribution of normalized counts for 2^0^-HPC/HSC-iPSC-18 over 2^0^-HPC/HSC-iPSC-16 in the TSS ± 2 kb regions from 332 overlapping genes and from 500 randomly selected genes was plotted (boxplot in R). The *horizontal dashed line* indicates no difference in the epigenetic modifications between 2^0^-HPC/HSC-iPSC-18 and 2^0^-HPC/HSC-iPSC-16; positive values indicate increased amounts of the modification in 2^0^-HPC/HSC-iPSC-18, and negative values indicate increased amounts of the modification in 2^0^-HPC/HSC-iPSC-16. *HPC/HSC* hematopoietic progenitor and stem cell, *iPSC* induced pluripotent stem cell

To this end, genome-wide analyses were performed to test whether a residual somatic memory exists in HPC/HSCs-iPSCs. First, the global memory of starting cells was compared between HPC/HSC-iPSCs and other origin-derived iPSCs; it was found that no global memory was detectable in the HPC/HSC-iPSCs based on our previous genome-wide sequencing analyses data [18]. We analyzed the global gene expression profiles of MEFs, adipose progenitor cells (APCs) and HPC/HSCs. The HPC/HSC-specific genes were identified through the comparison of HPC/HSCs with MEFs and APCs, two cell types that originate from mesenchymal stem cells, and are used as a reference for further comparisons in memory-related analysis. The differentially expressed genes in the two HPC/HSC-iPSC lines, the 2^0^-HPC/HSC-iPSC-16 (4 N-ON) and the 2^0^-HPC/HSC-iPSC-18 (4 N-OFF) line, were also screened, and the expression patterns of these differentially expressed genes were further analyzed and compared with the genes specifically expressed in HPC/HSCs to determine which iPSC line most closely resembled HPC/HSCs. In this analysis, 2910 genes were found to be specifically expressed in HPC/HSCs (1340 genes with high expression and 1570 genes with low expression). Interestingly, the 2^0^-HPC/HSC-iPSC-18 line more closely resembles HPC/HSCs than the 2^0^-HPC/HSC-iPSC-16 line in terms of gene expression patterns; notably, its gene expression pattern clusters with the HPC/HSC lowly expressed genes (Fig. 4a). We found 310 genes to be at least twofold more highly expressed in the 2^0^-HPC/HSC-iPSC-18 line than in the 2^0^-HPC/HSC-iPSC-16 line (Fig. 4b); likewise, 610 genes were found to be at least twofold more highly expressed in the 2^0^-HPC/HSC-iPSC-16 line than in the 2^0^-HPC/HSC-iPSC-18 line (Fig. 4c, top). Notably, among the 610 lowly expressed genes found in the 2^0^-HPC/HSC-iPSC-18 line, the expression of 332 genes overlapped with the gene expression pattern of the HPC/HSCs, indicating that the 2^0^-HPC/HSC-iPSC-18 line retained some transcriptional characteristics of the HPC/HSCs (Fig. 4c, top). Additionally, the most enriched GO term associated with the overlapping 332 genes indicated correlation of these genes with hematopoietic functions such as “vasculature development” and “blood vessel development” (Fig. 4c, bottom). Similarly, of the 310 highly expressed genes in the 2^0^-HPC/HSC-iPSC-18 line, 25 genes also overlapped with those of the HPC/HSCs, a greater number than that observed in the 2^0^-HPC/HSC-iPSC-16 line (13 genes; Fig. 4b). However, these 25 genes were not sufficient to reveal any enriched GO category. These findings demonstrate that minor transcriptional memory was retained in the 2^0^-HPC/HSC-iPSC-18 line, suggesting that incomplete reprogramming may occur in some 4 N-incompetent HPC/HSC-iPSCs.

To know whether the minor transcriptional memory was prevalent in iPSCs from other origins, two iPSC lines, 1^0^-MEF-iPSC-37 and 1^0^-MEF-iPSC-42, were also randomly selected to confirm whether similar minor transcriptional memory was retained in MEF-iPSCs. In agreement with our previous report [18], the 1^0^-MEF-iPSC-37 and 1^0^-MEF-iPSC-42 lines were indistinguishable at the level of genome-wide gene expression (Additional file 3: Figure S3A, B). However, neither minor transcriptional memory nor remarkable GO results were observed for these two iPSC lines, indicating that the observed transcriptional memory in the HPC/HSC-iPSC line occurred randomly and was not inherent to every iPSC line (Additional file 3: Figure S3C). Together, these results show that HPC/HSC-iPSCs and MEF-iPSCs derived from our sequential reprogramming system have no obvious memory of starting cells; nevertheless, minor residual transcriptional memory is retained in specific 4 N-incompetent HPC/HSC-iPSCs.

### H3K27me3 correlates with the minor transcriptional memory of HPC/HSC-iPSCs

To elucidate the reason for the minor transcriptional memory, epigenetic modifications near the TSS for these transcriptional memory-related genes were examined. Among the epigenetic modifications, 5mC, H3K4me2 and H3K4me3 showed no obvious difference in the TSS of these genes between 2^0^-HPC/HSC-iPSC-18 and 2^0^-HPC/HSC-iPSC-16 (Fig. 4d). However, intense H3K27me3 signals that deviate from random sampling were observed in 2^0^-HPC/HSC-iPSC-18 compared with 2^0^-HPC/HSC-iPSC-16, which may account for the repression of genes with minor transcriptional memory (Fig. 4d).

Collectively, the work presented here shows that HPC/HSCs are amenable to reprogramming through the use of a sequential reprogramming system into iPSCs with an unbiased hematopoietic differentiation capacity and no global epigenetic memory. Genome-wide analyses revealed that only a specific 4 N-incompetent HPC/HSC-iPSC line carried a minor transcriptional memory of starting cells, which had no influence on the differentiation capacity to hematopoietic progenitors and mature hematopoietic cells. Further analysis revealed that the aberrant distribution of H3K27me3 is associated with the observed minor transcriptional memory.

## Discussion

In the present study, HPC/HSC reprogramming was investigated, in terms of its molecular features and their effects on differentiation capacity. To the best of our knowledge, this study is the first demonstration that HPC/HSCs are amenable to be reprogrammed into iPSCs with unbiased hematopoietic differentiation capacity and no global epigenetic memory. However, a specific 4 N-incompetent HPC/HSC-iPSC line retained minor transcriptional memory of the starting cells, which had no influence on the hematopoietic differentiation capacity. Through genome-wide analysis, we found balanced occupancy of H3K4me3/H3K4me2 and H3K27me3 in HPC/HSC-specific genes, suggesting that histone modification transition is indispensable for HPC/HSC reprogramming. Furthermore, we discovered the correlation between the aberrant distribution of H3K27me3 and minor transcriptional memory.

The biosafety of iPSCs determines the future of cell transplantation-based regenerative medicine, and it is supposed that the quality of iPSCs can be improved by altering reprogramming strategies. It is noteworthy that totipotent iPSCs can be obtained by optimizing induction conditions [30, 31]. Moreover, the addition or substitution of specific reprogramming-related genes under traditional reprogramming conditions has been shown to give rise to more pluripotent iPSCs [32, 33]. The transcription factor-mediated cell fate reversion from somatic cells to pluripotent cells is accompanied by epigenetic transitions, in which the epigenetic state of the starting cells must be adjusted to a pluripotency compatible state [2]. A bivalent histone methylation structure in ESCs has been discovered to be associated with the poised state of cell lineage-specific genes [25]. Moreover, the performance of cell lineage-specific genes always correlates with the epigenetic memory observed in iPSCs [4, 5]. In the present study, it was found that HPC/HSC-specific genes possess a specific inverse occupancy of H3K4me2/H3K4me3 and H3K27me3 patterns, which may participate in the observed different epigenetic transition in transcription factor-mediated reprogramming of HPC/HSCs. Further elucidation of the role of the inverse occupancy of H3K4me2/H3K4me3 and H3K27me3 will undoubtedly shed light on epigenetic reprogramming.

The present genome-wide analyses found that minor transcriptional memory existed in one 4 N-incompoent iPSC line; however, the minor transcriptional memory had no influence on the hematopoietic lineage differentiation capacity, which was not completely consistent with the genome-wide epigenetic memory, as previously reported [4, 5]. A possible explanation for this discrepancy is the different results from other variations of these two reprogramming systems. That is, although the lentivirus vectors used here and in the previous study [4] are the same, the copy number of proviruses may be different in these two systems. In addition, the unique stoichiometry of OSKM in the HPC/HSC reprogramming observed here may also account for the quality of HPC/HSC-iPSCs, as previously reported [26].

Concerning the cause of epigenetic memory, several explanations have been proposed to answer this question. DNA methylation has been reported to be associated with the epigenetic memory observed in hematopoietic cell-derived iPSCs [4]. It has also been reported that low-passage iPSCs carry a significant epigenetic memory, and in vitro cell culture can effectively eliminate genome-wide epigenetic memory [5]. However, the low-passage iPSCs used here did not show a biased differentiation capacity to hematopoietic cells. In addition, miR-155 has been shown to be associated with epigenetic memory in hematopoietic cell-derived iPSCs [34]; however, a differential expression pattern of miR-155 was not observed in iPSCs derived from the previously established sequential reprogramming system [18]. Notably, the subtype of HPCs used in that study (CD133^+^) was also different from the one used here (c-kit^+^), in which miR-155 may not exert a dominant role [34].

A recent study revealed that the reprogramming process of HPC/HSCs activates a number of specific lncRNAs [29], which is consistent with a previous study from this research group [18]. Therefore, it is of interest to examine the correlation between lncRNAs and epigenetic memory. Notably, it has been found that the hematopoietic differentiation capability of hiPSCs can be divided into a commitment phase and a mature phase, which correlate with the expression of IGF2 and de novo methylation in reprogramming, respectively [35]. Although there were some variations in the expression of *Igf2* in the iPSC lines used here, no strict correlation between the expression of *Igf2* and hematopoietic differentiation capability was observed (data not shown). Nevertheless, in the current study it was found that the minor transcriptional memory in 4 N-OFF HPC/HSC-iPSCs was associated with the aberrant distribution of H3K27me3, which has recently been found to be correlated with the epigenetic reprogramming process in early embryos [36]. Future investigation of the role of H3K27me3 distribution in reprogramming will provide more information on the molecular barriers to reprogramming.

The availability of high-quality iPSCs with full pluripotency, no somatic memory and an unbiased differentiation capacity is ideal for the potential therapeutic use of iPSCs. The present study and our previous study [15] provide evidence that HPC/HSCs are amenable to reprogramming into high-quality iPSCs with full pluripotency, minor transcriptional memory and unbiased differentiation potential; however, somatic memory seems to be inherent to transcription factor-mediated reprogramming for the existence of minor transcriptional memory. Further studies on the reprogramming of HPC/HSCs or other progenitor cells will shed new light on reprogramming mechanisms and provide more alternatives to obtain high-quality iPSCs.

## Conclusions

HPC/HSCs undergo a unique reprogramming process under the sequential reprogramming system we established, which is different from that of MEFs. Genome-wide analyses revealed that there is no detectable epigenetic memory in HPC/HSC-iPSCs; nevertheless, very minor transcriptional memory was observed in one specific 4 N-incompetent type of iPSC, which resulted from the aberrant distribution of H3K27me3 in the region of the transcription start sites (TSS). However, the minor transcriptional memory had no impact on the differentiation of HPC/HSC-iPSCs into hematopoietic progenitors and mature hematopoietic cells.

## Additional files

Additional file 1: Figure S1. The expression of SSEA-1 in ESCs and iPSCs after 8 days of co-culture with OP9 stromal cells. In the first column, ESCs and iPSCs that were cultured on feeder cells for 8 days were isolated by SSC (side scatter) and SSEA-1. In the second column, ESCs or iPSCs that were co-cultured with OP9 stromal cells for 8 days were isolated by SSC and SSEA-1. *APC* adipose progenitor cell, *MEF* mouse embryonic fibroblast, *HPC/HSC* hematopoietic progenitor and stem cell*, iPSC* induced pluripotent stem cell, *SSC* side scatter. (TIF 2617 kb)
Additional file 2: Figure S2. Analysis of the differentiation potential of HPC/HSC-iPSCs into hematopoietic cells by flow cytometry. In the first column, cells were isolated based on size, as indicated by SSC and FSC (forward scatter). In the second column, GFP-negative cells were further selected. In the third column, c-Kit and Sca-1 double-positive (c-Kit^+^Sca-1^+^) cells were isolated. In the fourth column, c-Kit^+^Sca-1^+^ cells were further divided into CD34^+^ and CD34^-^ subpopulations. *APC* adipose progenitor cell, *MEF* mouse embryonic fibroblast, *HPC/HSC* hematopoietic progenitor and stem cell, *iPSC* induced pluripotent stem cell, *SSC* side scatter, *FSC* forward scatter, *APC* allophycocyanin, *PE-Cy7* phycoerythrin-Cy7, *CD* cluster of differentiation. (TIF 3169 kb)
Additional file 3: Figure S3. No transcriptional memory in MEF-iPSCs. (A) 1^0^-MEF-iPSC-37 was indistinguishable from 1^0^-MEF-iPSC-42 at the level of global gene expression. *Cor* Pearson correlation coefficient, *P P* value, *X, Y* log expression value. (B) No clustering of gene expression was observed in 1^0^-MEF-iPSC-37 and 1^0^-MEF-iPSC-42. (C) No functional GO term enrichment was observed between 1^0^-MEF-iPSC-37 and 1^0^-MEF-iPSC-42. *APC* adipose progenitor cell, *MEF* mouse embryonic fibroblast, *HPC/HSC* hematopoietic progenitor and stem cell, *iPSC* induced pluripotent stem cell. (TIF 3267 kb)

## Abbreviations

4 N: tetraploid complementation
APCs: adipose progenitor cells
BFU-E: erythrocyte
CFU: colony-forming unit
ChIP-Seq: chromatin immunoprecipitation sequencing
EB: embryonic body
ESCs: embryonic stem cells
FACS: fluorescence-activating cell sorting FBS, fetal bovine serum
FPKM: fragments per kilobase of exon per million fragments mapped
GEMM: granulocyte-erythrocyte-macrophage-megakaryocyte
GM: granulocyte-macrophage
GO: Gene Ontology
HPC: hematopoietic progenitor cell
HPC/HSC: hematopoietic progenitor and stem cell
HSC: hematopoietic stem cell
iPSC: induced pluripotent stem cell
M: macrophage
MeDIP: methylated DNA immunoprecipitation sequencing
MEF: mouse embryonic fibroblast
M-iPSCs: mesenchyme-derived iPSCs
MTG: monothioglycerol
RNA-Seq: RNA sequencing
SD: standard deviation
TSS: transcription start site

## References

[1] Takahashi K, Yamanaka S (2006). Induction of pluripotent stem cells from mouse embryonic and adult fibroblast cultures by defined factors. Cell.

[2] Hochedlinger K, Jaenisch R (2006). Nuclear reprogramming and pluripotency. Nature.

[3] O’Malley J, Skylaki S, Iwabuchi KA, Chantzoura E, Ruetz T, Johnsson A (2013). High-resolution analysis with novel cell-surface markers identifies routes to iPS cells. Nature.

[4] Kim K, Doi A, Wen B, Ng K, Zhao R, Cahan P (2010). Epigenetic memory in induced pluripotent stem cells. Nature.

[5] Polo JM, Liu S, Figueroa ME, Kulalert W, Eminli S, Tan KY (2010). Cell type of origin influences the molecular and functional properties of mouse induced pluripotent stem cells. Nat Biotechnol.

[6] Kim K, Zhao R, Doi A, Ng K, Unternaehrer J, Cahan P (2011). Donor cell type can influence the epigenome and differentiation potential of human induced pluripotent stem cells. Nat Biotechnol.

[7] Bar-Nur O, Russ HA, Efrat S, Benvenisty N (2011). Epigenetic memory and preferential lineage-specific differentiation in induced pluripotent stem cells derived from human pancreatic islet beta cells. Cell Stem Cell.

[8] Lister R, Pelizzola M, Kida YS, Hawkins RD, Nery JR, Hon G (2011). Hotspots of aberrant epigenomic reprogramming in human induced pluripotent stem cells. Nature.

[9] Ma H, Morey R, O’Neil RC, He Y, Daughtry B, Schultz MD (2014). Abnormalities in human pluripotent cells due to reprogramming mechanisms. Nature.

[10] Ohm JE, Mali P, Van Neste L, Berman DM, Liang L, Pandiyan K (2010). Cancer-related epigenome changes associated with reprogramming to induced pluripotent stem cells. Cancer Res.

[11] Giorgetti A, Montserrat N, Aasen T, Gonzalez F, Rodriguez-Piza I, Vassena R (2009). Generation of induced pluripotent stem cells from human cord blood using OCT4 and SOX2. Cell Stem Cell.

[12] Haase A, Olmer R, Schwanke K, Wunderlich S, Merkert S, Hess C (2009). Generation of induced pluripotent stem cells from human cord blood. Cell Stem Cell.

[13] Eminli S, Foudi A, Stadtfeld M, Maherali N, Ahfeldt T, Mostoslavsky G (2009). Differentiation stage determines potential of hematopoietic cells for reprogramming into induced pluripotent stem cells. Nat Genet.

[14] Guo S, Zi X, Schulz VP, Cheng J, Zhong M, Koochaki SH (2014). Nonstochastic reprogramming from a privileged somatic cell state. Cell.

[15] Gao S, Tao L, Hou X, Xu Z, Liu W, Zhao K (2016). Genome-wide gene expression analyses reveal unique cellular characteristics related to the amenability of HPC/HSCs into high-quality induced pluripotent stem cells. Stem Cell Res Ther.

[16] Gao S, Zheng C, Chang G, Liu W, Kou X, Tan K (2015). Unique features of mutations revealed by sequentially reprogrammed induced pluripotent stem cells. Nat Commun.

[17] Nakano T (1996). In vitro development of hematopoietic system from mouse embryonic stem cells: a new approach for embryonic hematopoiesis. Int J Hematol.

[18] Chang G, Gao S, Hou X, Xu Z, Liu Y, Kang L (2014). High-throughput sequencing reveals the disruption of methylation of imprinted gene in induced pluripotent stem cells. Cell Res.

[19] Langmead B, Trapnell C, Pop M, Salzberg SL (2009). Ultrafast and memory-efficient alignment of short DNA sequences to the human genome. Genome Biol.

[20] Trapnell C, Pachter L, Salzberg SL (2009). TopHat: discovering splice junctions with RNA-Seq. Bioinformatics.

[21] Roberts A, Pimentel H, Trapnell C, Pachter L (2011). Identification of novel transcripts in annotated genomes using RNA-Seq. Bioinformatics.

[22] Zhang Y, Liu T, Meyer CA, Eeckhoute J, Johnson DS, Bernstein BE (2008). Model-based analysis of ChIP-Seq (MACS). Genome Biol.

[23] Xu H, Handoko L, Wei X, Ye C, Sheng J, Wei CL (2010). A signal-noise model for significance analysis of ChIP-seq with negative control. Bioinformatics.

[24] da Huang W, Sherman BT, Lempicki RA (2009). Systematic and integrative analysis of large gene lists using DAVID bioinformatics resources. Nat Protoc.

[25] Bernstein BE, Mikkelsen TS, Xie X, Kamal M, Huebert DJ, Cuff J (2006). A bivalent chromatin structure marks key developmental genes in embryonic stem cells. Cell.

[26] Carey BW, Markoulaki S, Hanna JH, Faddah DA, Buganim Y, Kim J (2011). Reprogramming factor stoichiometry influences the epigenetic state and biological properties of induced pluripotent stem cells. Cell Stem Cell.

[27] Stadtfeld M, Apostolou E, Akutsu H, Fukuda A, Follett P, Natesan S (2010). Aberrant silencing of imprinted genes on chromosome 12qF1 in mouse induced pluripotent stem cells. Nature.

[28] Liu L, Luo GZ, Yang W, Zhao X, Zheng Q, Lv Z (2010). Activation of the imprinted Dlk1-Dio3 region correlates with pluripotency levels of mouse stem cells. J Biol Chem.

[29] Kim DH, Marinov GK, Pepke S, Singer ZS, He P, Williams B (2015). Single-cell transcriptome analysis reveals dynamic changes in lncRNA expression during reprogramming. Cell Stem Cell.

[30] Zhao XY, Li W, Lv Z, Liu L, Tong M, Hai T (2009). iPS cells produce viable mice through tetraploid complementation. Nature.

[31] Kang L, Wang J, Zhang Y, Kou Z, Gao S (2009). iPS cells can support full-term development of tetraploid blastocyst-complemented embryos. Cell Stem Cell.

[32] Jiang J, Lv W, Ye X, Wang L, Zhang M, Yang H (2013). Zscan4 promotes genomic stability during reprogramming and dramatically improves the quality of iPS cells as demonstrated by tetraploid complementation. Cell Res.

[33] Chen J, Gao Y, Huang H, Xu K, Chen X, Jiang Y (2015). The combination of Tet1 with Oct4 generates high-quality mouse-induced pluripotent stem cells. Stem Cells.

[34] Vitaloni M, Pulecio J, Bilic J, Kuebler B, Laricchia-Robbio L, Izpisua Belmonte JC (2014). MicroRNAs contribute to induced pluripotent stem cell somatic donor memory. J Biol Chem.

[35] Nishizawa M, Chonabayashi K, Nomura M, Tanaka A, Nakamura M, Inagaki A (2016). Epigenetic variation between human induced pluripotent stem cell lines is an indicator of differentiation capacity. Cell Stem Cell.

[36] Zheng H, Huang B, Zhang B, Xiang Y, Du Z, Xu Q (2016). Resetting epigenetic memory by reprogramming of histone modifications in mammals. Mol Cell.

