# Supplementary figures and images for: Integrated analysis of hematopoietic differentiation outcomes and molecular characterization reveals unbiased differentiation capacity and minor transcriptional memory in HPC/HSC-iPSCs
Source: Stem Cell Res Ther. 2017 Jan 23;8:13. doi: 10.1186/s13287-016-0466-1 (PMC5259886; doi:10.1186/s13287-016-0466-1)

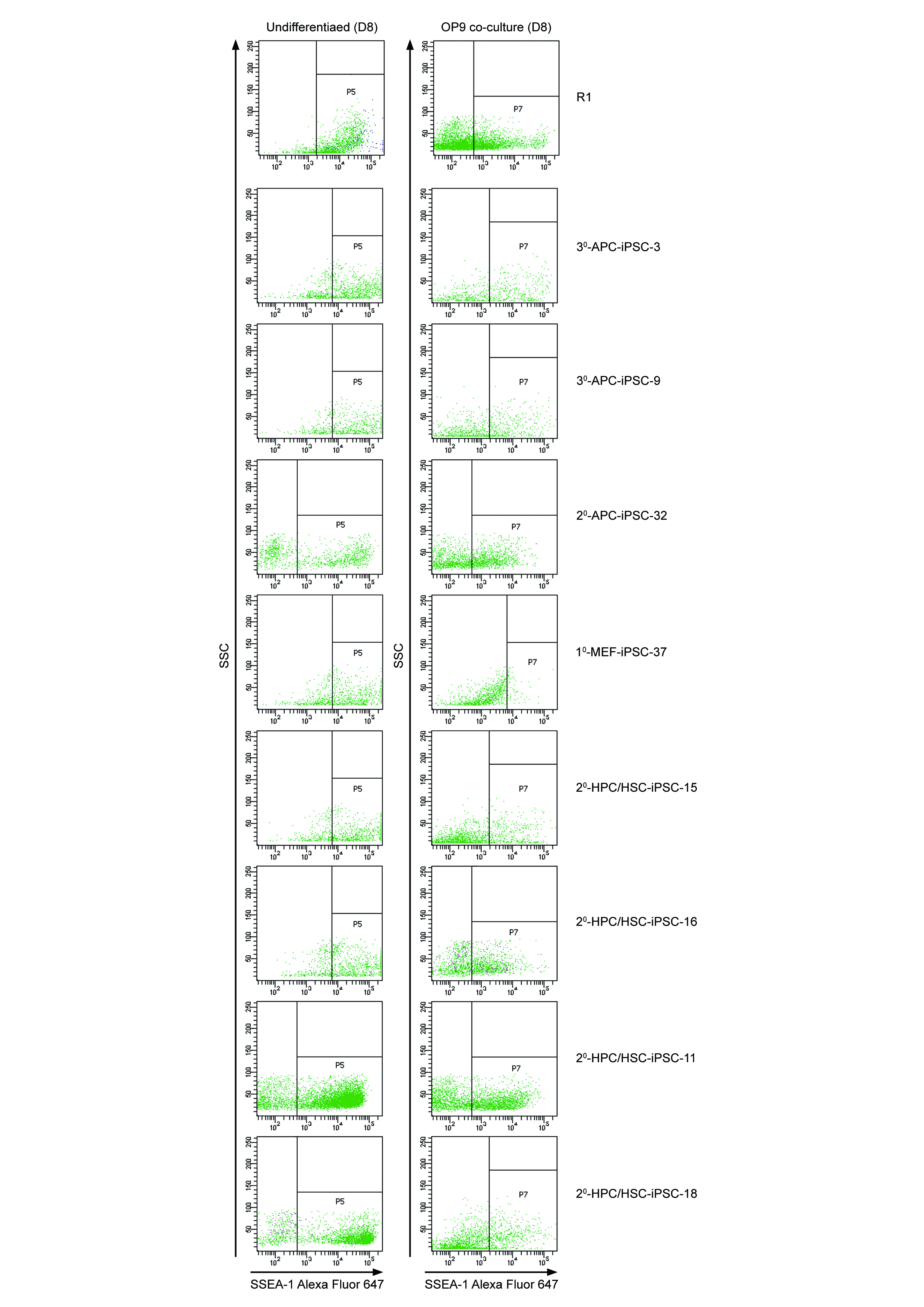

Supplement: Additional file 1: Figure S1. — The expression of SSEA-1 in ESCs and iPSCs after 8 days of co-culture with OP9 stromal cells. In the first column, ESCs and iPSCs that were cultured on feeder cells for 8 days were isolated by SSC (side scatter) and SSEA-1. In the second column, ESCs or iPSCs that were co-cultured with OP9 stromal cells for 8 days were isolated by SSC and SSEA-1. APC adipose progenitor cell, MEF mouse embryonic fibroblast, HPC/HSC hematopoietic progenitor and stem cell, iPSC induced pluripotent stem cell, SSC side scatter. (TIF 2617 kb) [file 13287_2016_466_MOESM1_ESM.tif]

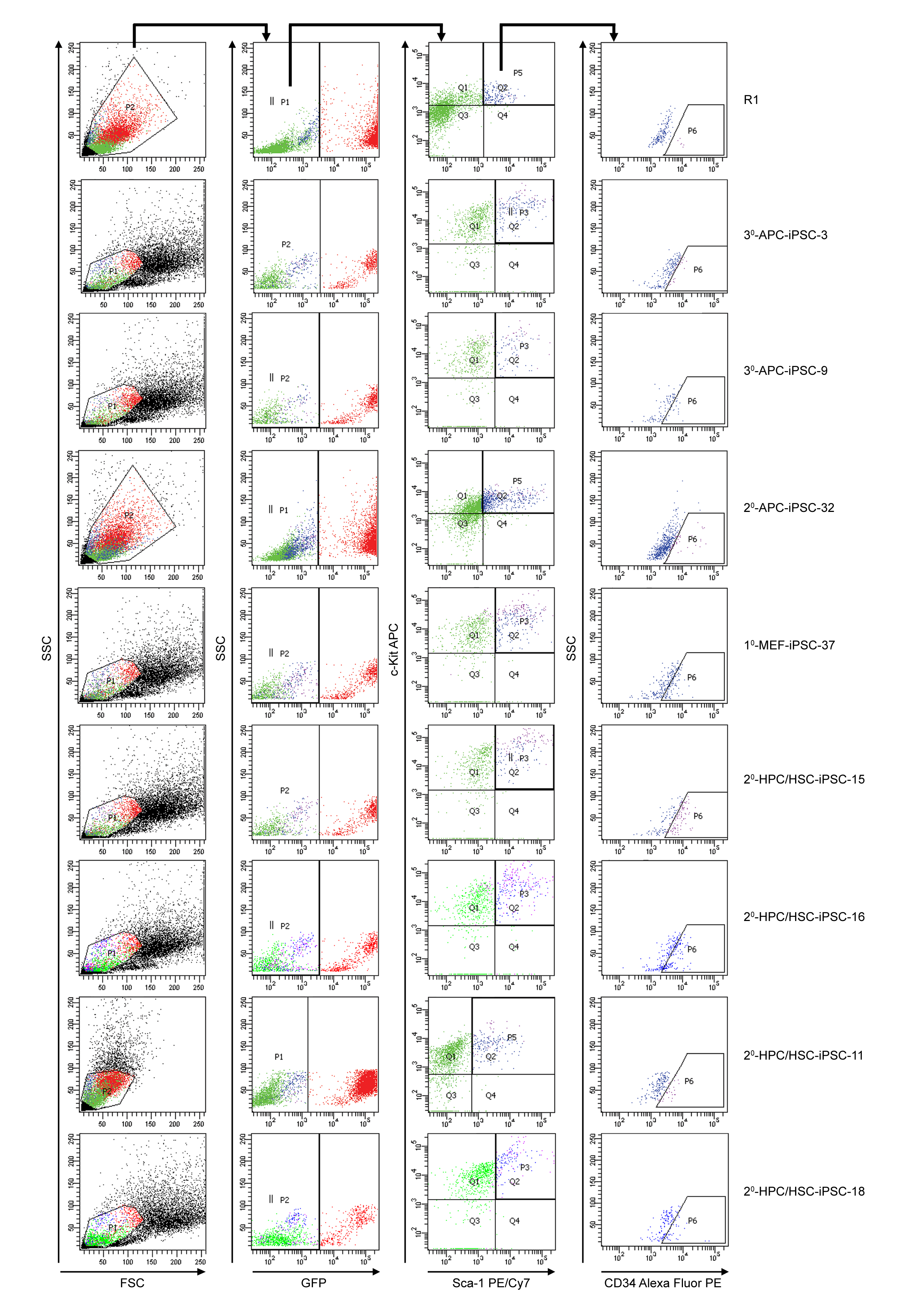

Supplement: Additional file 2: Figure S2. — Analysis of the differentiation potential of HPC/HSC-iPSCs into hematopoietic cells by flow cytometry. In the first column, cells were isolated based on size, as indicated by SSC and FSC (forward scatter). In the second column, GFP-negative cells were further selected. In the third column, c-Kit and Sca-1 double-positive (c-Kit+Sca-1+) cells were isolated. In the fourth column, c-Kit+Sca-1+ cells were further divided into CD34+ and CD34- subpopulations. APC adipose progenitor cell, MEF mouse embryonic fibroblast, HPC/HSC hematopoietic progenitor and stem cell, iPSC induced pluripotent stem cell, SSC side scatter, FSC forward scatter, APC allophycocyanin, PE-Cy7 phycoerythrin-Cy7, CD cluster of differentiation. (TIF 3169 kb) [file 13287_2016_466_MOESM2_ESM.tif]

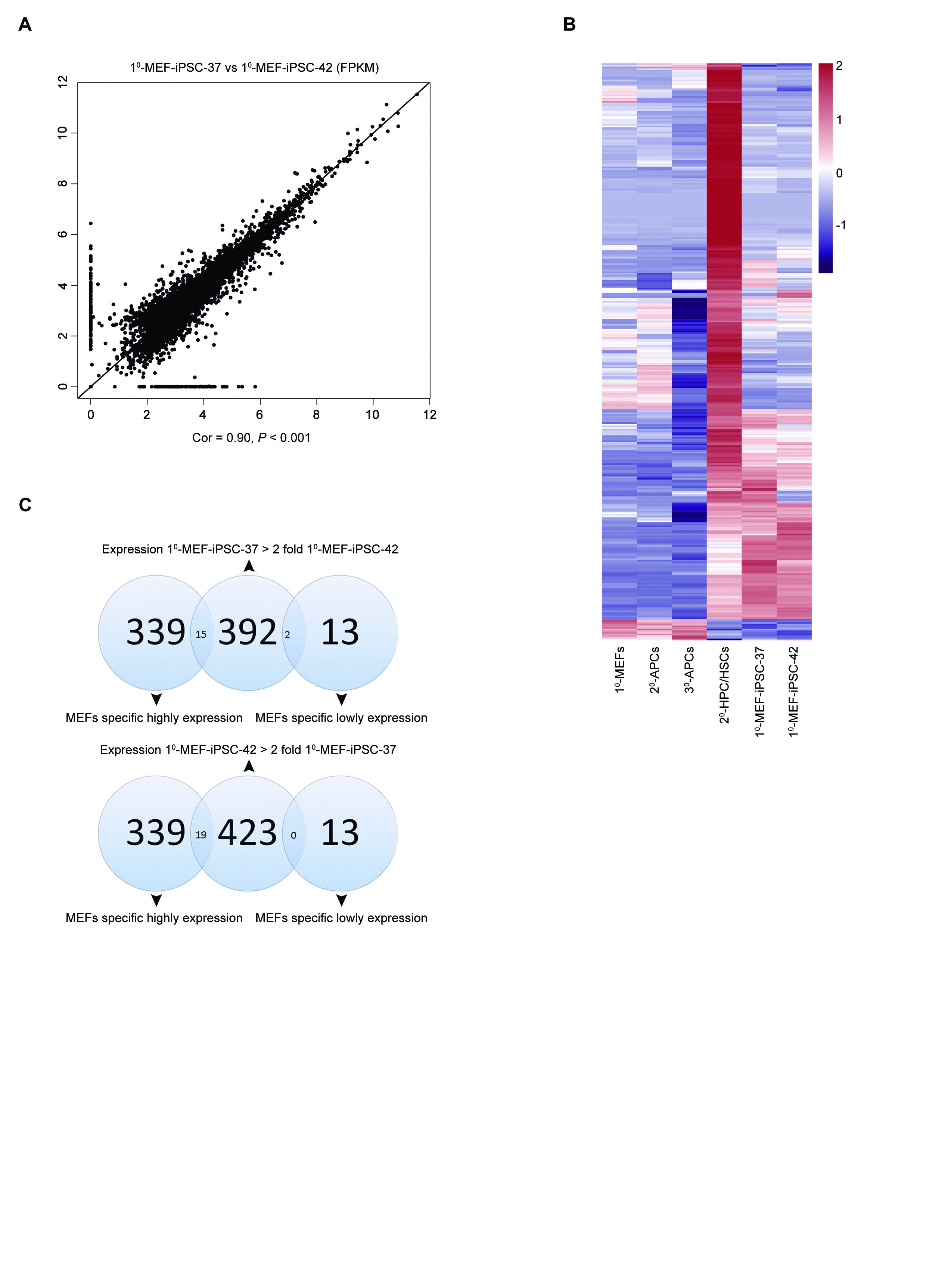

Supplement: Additional file 3: Figure S3. — No transcriptional memory in MEF-iPSCs. (A) 10-MEF-iPSC-37 was indistinguishable from 10-MEF-iPSC-42 at the level of global gene expression. Cor Pearson correlation coefficient, P P value, X, Y log expression value. (B) No clustering of gene expression was observed in 10-MEF-iPSC-37 and 10-MEF-iPSC-42. (C) No functional GO term enrichment was observed between 10-MEF-iPSC-37 and 10-MEF-iPSC-42. APC adipose progenitor cell, MEF mouse embryonic fibroblast, HPC/HSC hematopoietic progenitor and stem cell, iPSC induced pluripotent stem cell. (TIF 3267 kb) [file 13287_2016_466_MOESM3_ESM.tif]
